# Successful Treatment of Cutaneous Larva Migrans With Combined Albendazole and Ivermectin Therapy: A Report of Two Cases From Sudan

**DOI:** 10.7759/cureus.64665

**Published:** 2024-07-16

**Authors:** Mahdi Shamad, Nawaf Al-Mutairi

**Affiliations:** 1 College of Medicine, University of Bahri, Khartoum, SDN; 2 Faculty of Medicine, Kuwait University, Kuwait City, KWT

**Keywords:** hookworm, helminthic infection, ivermectin, albendazole, treatment, larva migrans, creeping eruption

## Abstract

Cutaneous larva migrans (CLM), caused by third-stage filariform larvae of cat and dog hookworms, presents as pruritic, serpiginous tracks upon skin penetration by larvae from contaminated soil. Herein, we report the successful treatment of two CLM patients using albendazole and ivermectin combination therapy. A 42-year-old man from Kordofan and a 38-year-old man from White Nile State presented with characteristic lesions on their lower extremities, resolving completely within one week post-treatment without recurrence. This report highlights the potential of combined albendazole-ivermectin therapy in managing CLM amid emerging antihelminthic resistance, suggesting that its broader application warrants further investigation.

## Introduction

Cutaneous larva migrans (CLM), known as creeping eruption, is a distinctive parasitic dermatologic condition caused by the migration of the third-stage filariform larvae of dog and cat hookworms, primarily *Ancylostoma braziliense* and occasionally *Ancylostoma caninum*. Characterized by intensely pruritic, serpiginous cutaneous tracks, CLM results when larvae from contaminated soil penetrate the skin and migrate through the epidermis. First identified in 1928 by White and Dove, CLM remains prevalent globally in tropical and subtropical regions, including Africa, Southeast Asia, Latin America, the Caribbean, and the Southwestern United States [1]. While typically self-limiting, the condition warrants treatment due to significant discomfort and a potential secondary bacterial infection [2].

Systemic administration of albendazole is a common treatment for CLM, although recurrence can occur [3]. Ivermectin is another option; however, rising helminthic resistance to both drugs underscores the need for novel therapeutic strategies [4]. Here, we detail the successful treatment of two Sudanese patients with the albendazole and ivermectin combination, chosen for their diverse mechanisms of action and established efficacy against parasitic infections, highlighting clinical outcomes and therapeutic considerations.

## Case presentation

Case 1

**Figure 1:**
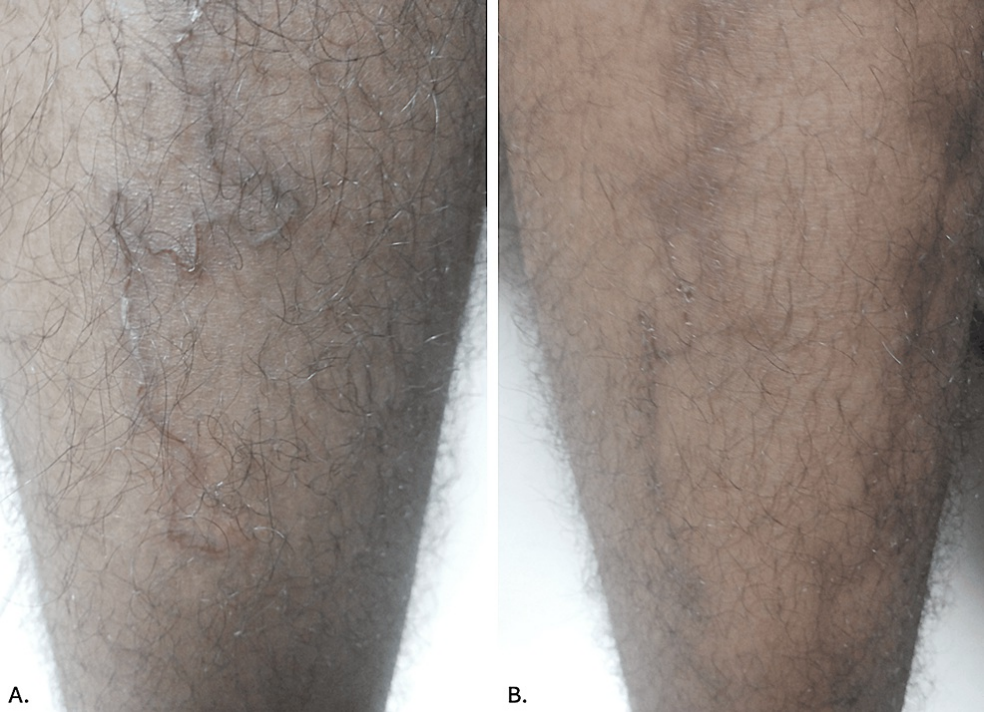
A. Linear, erythematous, serpiginous track on the anterior aspect of the right shin; B. Complete resolution one week after therapy, with only the presence of post-inflammatory hyperpigmentation.

A 42-year-old man from Kordofan State, Sudan, a farmer by occupation, presented to the dermatology outpatient department with complaints of a pruritic, red, linear eruption over the shin of his right leg. The lesions had been present for three weeks, preceded by intense pruritus in the affected area. He reported no past history of similar lesions, and his family history was nonsignificant. Cutaneous examination revealed a single erythematous superficial lesion over the anterior aspect of the right leg (Figure 1A), with no similar lesions elsewhere on his body. The routine blood investigations showed no eosinophilia. CLM was diagnosed based on clinical presentation. He was prescribed 400 mg of albendazole once daily for three days and a single 12 mg dose of ivermectin, leading to complete clinical resolution at the one-week follow-up visit (Figure 1B). The patient was followed up for an additional three weeks, during which no recurrence of lesions was observed.

Case 2

**Figure 2:**
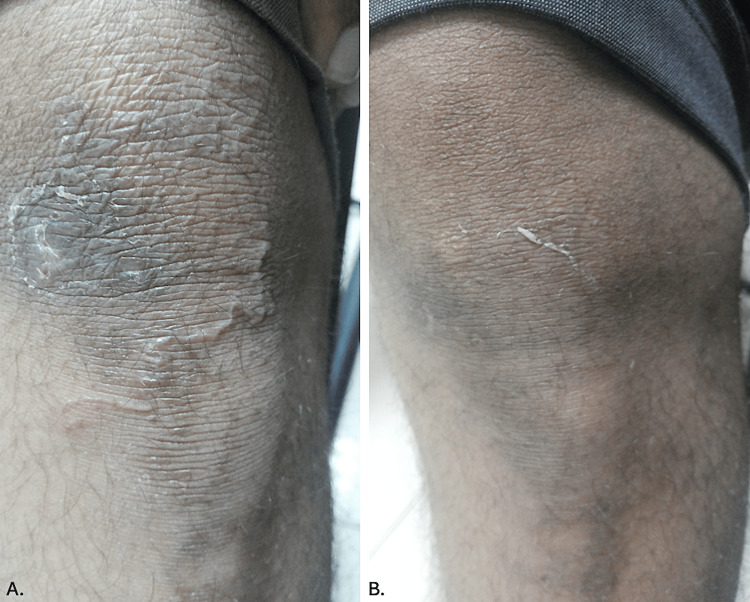
A. Erythematous, serpiginous lesions of cutaneous larva migrans on the anterior aspect of the right knee; B. Complete resolution seven days after combination therapy with albendazole and ivermectin.

A 38-year-old man from White Nile State, Sudan, working as a farmer, presented to the dermatology outpatient department with similar complaints of erythematous, pruritic, and linear eruption across his right knee for a duration of two weeks. These were preceded by mild itching and discomfort at the location. The patient reported no prior history of similar lesions, and there was no family history of similar lesions. The clinical examination revealed a solitary, serpiginous, and erythematous lesion on the anterior aspect of the right knee (Figure 2A). Investigations were normal, and treatment with a combination of albendazole and ivermectin resulted in complete clinical resolution within one week (Figure 2B). The patient maintained communication with our team for an additional three weeks, during which no recurrence of lesions was observed.

## Discussion

CLM predominantly occurs in tropical and subtropical climates, where conditions favor the survival and transmission of hookworm larvae [1]. Cases have been recorded by visitors from cold climate zones who have recently visited an endemic nation.

The life cycle of the parasite responsible for CLM begins when eggs from animal feces are deposited in warm, moist soil and hatch into larvae. Upon contact with human skin, third-stage larvae penetrate and infect the new host. Unable to develop in human hosts, larvae remain restricted to the epidermis, migrating a few millimeters daily. As demonstrated in our cases, lesions typically appear on the lower extremities but can occur elsewhere based on the person's exposure to contaminated soil. Although eruptions typically last between two and eight weeks, they can persist for years in some cases [5].

While CLM is commonly diagnosed based on clinical presentation and exposure history, histopathologic examination can confirm the diagnosis, revealing larva in the epidermis with spongiotic dermatitis and intraepidermal vesicles that primarily contain a mixed acute inflammatory infiltrate of eosinophils [6]. Systemic manifestations such as lung infiltrates, elevated immunoglobulin E (IgE) levels, and peripheral eosinophilia (Löffler syndrome) are rarely observed [7]. There was no evidence of pulmonary involvement or eosinophilia in the presented cases, and the diagnosis was made clinically based on characteristic lesions and endemic exposure.

Albendazole and ivermectin are the mainstays of CLM treatment due to their broad antiparasitic activity. Albendazole is typically prescribed at a dose of 400 mg taken orally with fatty meals for three days. However, to address cases of recurrence and partial remission, extending the treatment duration to seven days has shown promising results [8]. Ivermectin is administered as a single 12 mg dose. Research comparing the therapeutic efficaciousness of albendazole and ivermectin for the management of CLM has demonstrated a preference for the latter [3]. Albendazole disrupts parasite microtubule formation, impairing nutrient uptake and motility, while ivermectin induces paralysis through chloride channel hyperpolarization [9]. Despite the known efficacy of these agents as a standalone treatment, rising reports of helminthic resistance necessitate novel treatment strategies [4,10].

The co-administration of ivermectin and albendazole has been safely and effectively used in lymphatic filariasis since the 1980s, and several studies have demonstrated its usefulness in other soil-transmitted helminth infections [11]. Further, previous studies on other helminths have shown that combination therapy has a comparable safety profile as monotherapy with either drug [11]. However, the efficacy of this combination in CLM remains unexplored. The combined use of albendazole and ivermectin, as demonstrated in the presented cases, leverages their complementary mechanisms to enhance therapeutic outcomes, potentially reducing resistance development. Moreover, combination treatment offers the advantage of reducing the treatment duration compared to the seven-day albendazole regimen.

## Conclusions

The successful treatment of two Sudanese patients with CLM using a combination of albendazole and ivermectin demonstrates the potential efficacy of this therapeutic approach. Both patients experienced rapid and complete resolution of symptoms, with no recurrence during follow-up. This combination therapy may offer an effective alternative amid rising reports of antihelminthic resistance. Given the complementary mechanisms of action and the reduced treatment duration, further investigation into this combined approach is warranted to confirm its broader applicability and effectiveness in managing CLM.
